# Evaluation of Fibrin-Based Interpenetrating Polymer Networks as Potential Biomaterials for Tissue Engineering

**DOI:** 10.3390/nano7120436

**Published:** 2017-12-10

**Authors:** Olfat Gsib, Jean-Luc Duval, Mathieu Goczkowski, Marie Deneufchatel, Odile Fichet, Véronique Larreta-Garde, Sidi Ahmed Bencherif, Christophe Egles

**Affiliations:** 1 Laboratoire de BioMécanique et de BioIngénierie (BMBI) UMR CNRS 7388, Sorbonne Universités, Université de Technologie of Compiègne (UTC), 60200 Compiègne, France; 2 Equipe de Recherche sur les Relations Matrice Extracellulaire Cellules (Errmece), Institut des Matériaux, Université de Cergy-Pontoise, 95000 Cergy-Pontoise, France; 3 Laboratoire de Physicochimie des Polymères et des Interfaces (LPPI), Institut des Matériaux, Université de Cergy-Pontoise, 95000 Cergy-Pontoise, France; 4 School of Engineering and Applied Sciences, Harvard University, Cambridge, MA 02138, USA; 5 Department of Chemical Engineering, Northeastern University, 360 Huntington Avenue, Boston, MA 02215, USA

**Keywords:** interpenetrating polymer networks, fibrin, polyethylene oxide, serum albumin, fibrous hydrogel, biocompatibility, organotypic culture, tissue engineering, biomaterials

## Abstract

Interpenetrating polymer networks (IPNs) have gained great attention for a number of biomedical applications due to their improved properties compared to individual components alone. In this study, we investigated the capacity of newly-developed naturally-derived IPNs as potential biomaterials for tissue engineering. These IPNs combine the biologic properties of a fibrous fibrin network polymerized at the nanoscale and the mechanical stability of polyethylene oxide (PEO). First, we assessed their cytotoxicity in vitro on L929 fibroblasts. We further evaluated their biocompatibility ex vivo with a chick embryo organotypic culture model. Subcutaneous implantations of the matrices were subsequently conducted on nude mice to investigate their biocompatibility in vivo. Our preliminary data highlighted that our biomaterials were non-cytotoxic (viability above 90%). The organotypic culture showed that the IPN matrices induced higher cell adhesion (across all the explanted organ tissues) and migration (skin, intestine) than the control groups, suggesting the advantages of using a biomimetic, yet mechanically-reinforced IPN-based matrix. We observed no major inflammatory response up to 12 weeks post implantation. All together, these data suggest that these fibrin-based IPNs are promising biomaterials for tissue engineering.

## 1. Introduction

Over the past few decades, tissue engineering has made great progress, which has resulted in developing a large variety of polymeric scaffolds such as hydrogels [1,2,3,4,5,6,7]. Hydrogels are polymeric crosslinked networks with a high water content that can mimic the extracellular matrix of biological soft tissues [8]. They provide a three-dimensional architecture suitable to direct cellular behavior and to promote cell interactions [9,10,11].

A large number of hydrogels are fabricated with naturally-derived polymers in order to enhance their degradability, biocompatibility and bioactivity [9,12]. However, these hydrogels usually present poor mechanical properties [13,14,15]. For instance, fibrin-based hydrogels, which are widely used in tissue engineering, have inadequate mechanical performance, especially at the physiological concentration (2–5 mg/mL) where they quickly degrade, thus limiting their clinical applications [15,16,17,18]. However, fibrin itself presents several advantages and due to its key role in wound healing [15,19], fibrin can serve as a provisional biologically-active backbone to promote cell adhesion, migration and proliferation. Additionally, fibrin precursors, thrombin and fibrinogen, can be easily isolated from patients’ blood, making customizable treatments possible [15,20].

Several strategies have been used in order to improve the mechanical properties of fibrin hydrogels. Those techniques are based on fine-tuning various polymerization parameters such as pH, calcium or the concentration of pre-polymer precursors [21,22,23]. The resulting fibrin hydrogels usually display different fibrous structures and enhanced mechanical properties. However, fibrin networks synthetized under physiological conditions are required for cell culture as they closely mimic the in vivo environment. Another approach consists in bringing mechanical strength to fibrin networks by associating them with synthetic polymer networks. Yet, these blends are seen to lead to a macroscopic phase separation [24].

In recent years, a new approach has been developed to improve the mechanical performance of fibrin-based hydrogels and consists of combining fibrin networks with natural or synthetic networks to form interpenetrating polymer networks (IPNs). IPNs are described as “a combination of two or more polymers in network form, with at least one of them is synthetized and/or cross-linked in the presence of the other(s) but not covalently bonded to each other and which cannot be separated unless chemical bonds are broken” [25,26,27,28]. Several studies reported the combination of fibrin networks with other polymer networks in IPNs. Fibrin was associated with hyaluronic acid in IPNs to form new hydrogels with improved mechanical properties [25,29]. Fibrin-alginate IPNs were synthetized to provide a dynamic matrix for the growth of ovarian follicle cells [30]. IPNs combining collagen with fibrin were developed in order to create biomaterials for cardio tissue engineering that yield better mechanical strength compared to each component alone [31]. However, these systems combined fibrin with other naturally-derived polymers, therefore, limiting the extent of their mechanical enhancement. The combination of fibrin with a synthetic polymer would allow more flexibility to fine-tune the physical properties of fibrin-containing IPNs. Polyethylene oxide (PEO) and polyethylene glycol (PEG) are bioinert synthetic polymers that present versatile physical properties [32,33] combined with highly hydrophilic characteristics making them very appealing for a number of biomedical applications [34]. Many medical devices incorporating PEO have been already approved by the food and drug administration [8]. Other polymers such as polyvinyl alcohol (PVA), with its semi-crystalline and hygroscopic properties, have also shown their potentials for various biomedical uses [35,36,37,38].

In previous studies, we designed IPNs combining a fibrin network with PEO or PVA networks. These materials presented good mechanical properties, being self-supported, non-retractable, re-hydratable and non-cytotoxic [24,39]. These IPNs combined the biological properties of a fibrous fibrin network polymerized enzymatically under physiological conditions with the suitable mechanical properties of a synthetic polymer network formed through a photopolymerization process. We also turned PVA-fibrin-based IPNs into materials with tunable degradability by incorporating enzymatically-degradable regions within the polymer backbone [40].

In the present study, we adapted the protocol for PEO by combining the polymer with enzymatically-degradable serum albumin functionalized with methacrylate residues (SAm). Albumins such as bovine serum albumin (BSA) and human serum albumin (HSA) have found a wide range of biomedical applications partly due to their good biocompatibility, their degradability, their stability in the pH range of 4–9 and their ready commercial availability at affordable costs [41,42]. Furthermore, several studies showed the key role of albumin in the fibrin network structure and degradability [43,44,45].

In this work, we investigated different techniques to evaluate their cytotoxicity and biocompatibility. An in vitro cytotoxic assay based on a derivative of the 3-(4,5-dimethylthiazol-2-yl)-2,5-diphenyltetrazolium bromide (MTT) assay was performed to first assess their cytocompatibility [46,47,48]. This assay is described in the ISO-10993 standards (Part 5), which are standards for evaluating the biocompatibility of medical devices [49].

Other techniques such as ex vivo and in vivo tests were required to investigate further the biocompatibility of our biomaterials. A chick embryo organotypic culture model was used to evaluate the biocompatibility ex vivo. Organotypic culture, developed by Wolff and Haven in 1952, has already been used in several studies to determine the biocompatibility of biomaterials [50,51,52]. This method gives a quick estimate of parameters characterizing the cell behavior such as cell adhesion, proliferation or migration, which can further help to choose the final application of the biomaterials of interest. In our study, we isolated skin, intestine, gonads, blood vessels, cornea and brain fragment organs from the chick embryos and screened at the same time different potential biomedical applications.

Subcutaneous implantations on the backs of five-week-old male nude mice were eventually performed to evaluate the biocompatibility in vivo. We thus assessed potential irritation and inflammation responses, the cellular infiltration and the integration of our matrices within the surrounding tissues.

## 2. Results

### 2.1. Synthesis of Fibrin-Based IPN Hydrogels

**Figure 1:**
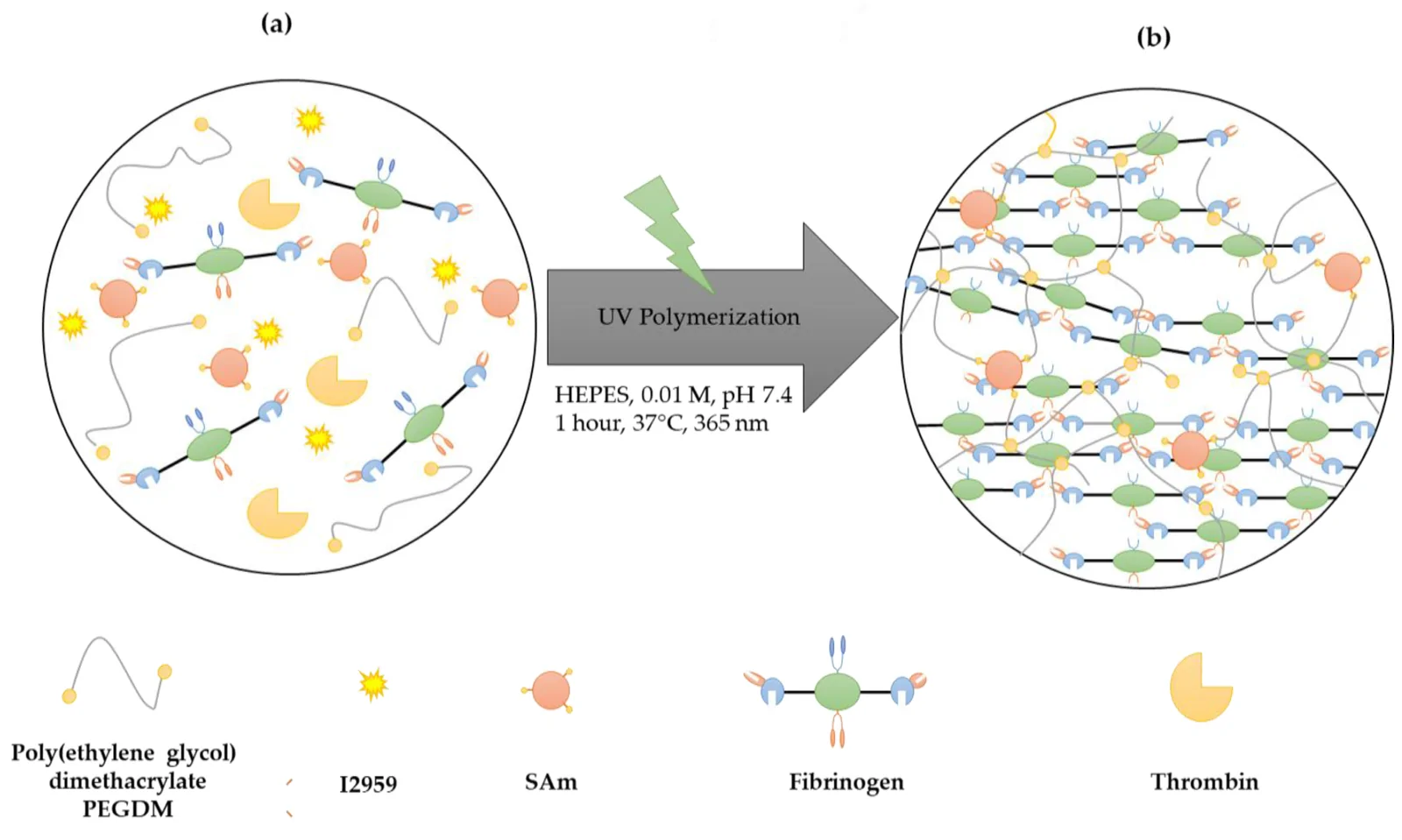
Scheme of interpenetrating polymer network (IPN) synthesis. (**a**) All the precursors are mixed together at the same time; (**b**) fibrin/polyethylene oxide (PEO)-serum albumin functionalized with methacrylate residues (SAm)-based IPNs are obtained after UV exposure (1.16 mW/cm^2^, 365 nm) for 1 h at 37 °C.

Fibrin-based IPN hydrogels (PEO(5%)coHSAm(5%)fb(0.5%) IPNs) combine a fibrin network formed under physiological conditions by enzymatic means with a PEO-SAm co-network synthetized simultaneously via free radical copolymerization (Figure 1): Human serum albumin was first modified with methacrylate residues (HSAm). The fibrin (fb) precursors (fibrinogen (0.5% *w*/*v*) and thrombin (0.2 U/mL) in the presence of calcium and sodium chloride buffer) were then mixed with PEO-SAm co-network precursors (poly(ethylene glycol) dimethacrylate (PEGDM, 5% *v*/*v*), SAm (5% *w*/*v*)) and the photo-initiator irgacure 2959 (I2959) in 2-[4-(2-Hydroxyethyl)-1-piperazine]ethanesulfonic acid (HEPES) buffer (Figure 1a). The mixture was exposed to UV light for initiating the free radical copolymerization (1.16 mW/cm^2^, 365 nm) leading to an IPN architecture (Figure 1b). Additional IPNs were also synthesized, i.e., IPNs either without SAm (PEO(10%)fb(0.5%) IPNs) or without PEO (HSAm(10%)fb(0.5%) IPNs), and used as controls for cytotoxic assays.

### 2.2. Cytocompatibility of IPN Hydrogels

As biomaterials are intended to be in contact with living tissues, it is crucial to evaluate as a first step their biocompatibility, which is a compromise between the best performance of the material in a biologic context and the least harmful host reaction in this biological situation [53]. To assess the biocompatibility of our various IPNs, we first realized the in vitro cytotoxic assay following the ISO-10993 guidelines Part 5, the standards for the biological evaluation of medical devices [49].

**Figure 2:**
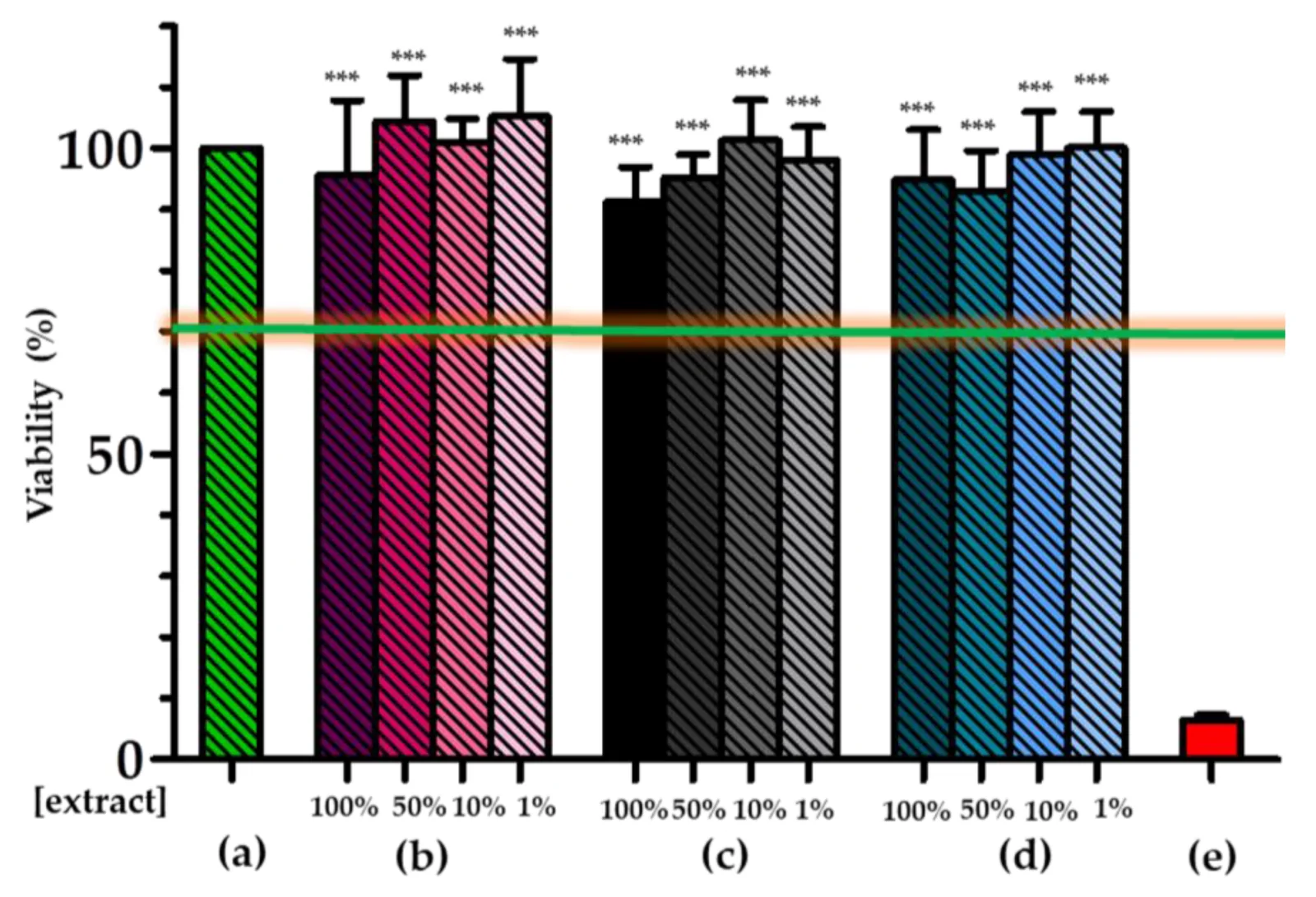
Viability of cells exposed to IPN extraction vehicles. An 3-(4,5-dimethylthiazol-2-yl)-5-(3-carboxymethoxyphenyl)-2-(4-sulfophenyl)-2H-tetrazolium, inner salt (MTS) cytotoxic assay was performed on cells cultured with IPN extraction vehicles from the three investigated materials and controls: non-treated cells (negative cytotoxic control) (**a**), HSAm(10%)fb(0.5%) IPNs (**b**), PEO(5%)coHSAm(5%)fb(0.5%) IPNs (**c**), PEO(10%)fb(0.5%) (**d**) IPNs and latex group (positive cytotoxic control) (**e**). Various extract concentrations were screened for IPN materials varying from 1–100%. Values represent the mean and SD (*n* = 5). Differences between groups were statistically significant. Data were normalized with respect to the non-treated controls (**a**) and analyzed using one-way ANOVA, *** *p* ≤ 0.0001.

L929 cells, which were cultured in contact with the extraction vehicles (the complete medium in which the IPN samples had been agitated during one day) of the PEO(5%)coHSAm(5%)fb(0.5%) IPNs and the two other control IPN compositions, were viable, as shown in Figure 2.

The viability of L929 remained higher than 90%, largely above a 70% viability threshold set in the ISO-10993 Part 5 standard, for which a material is considered to be non-cytotoxic [49].

**Figure 3:**
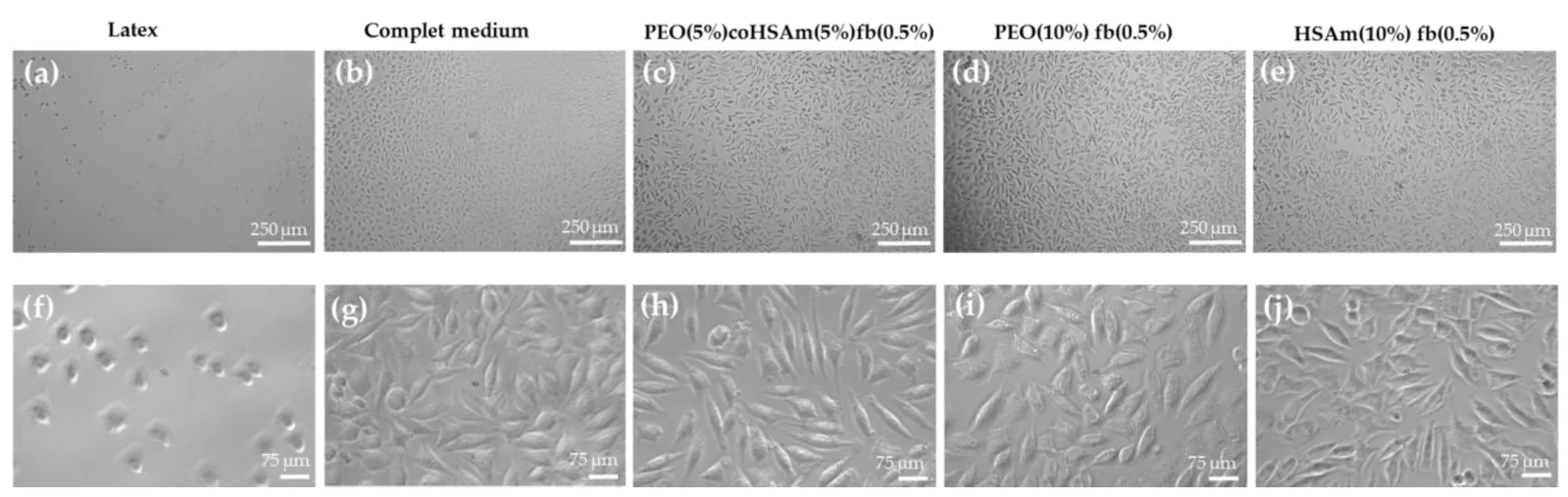
Morphology of cells exposed to IPN extracts. Phase contrast images of L929 cells three days following the cytotoxic assay, with magnifications (×10) (**a**–**e**) and (×40) (**f**–**j**). L929 cells cultured with: (**a**,**f**) extracts from latex (positive control for cytotoxicity); (**b**,**g**) no extract (negative control for cytotoxicity); (**c**,**h**) extracts from PEO(5%)coHSAm(5%)fb(0.5%) IPNs; (**d**,**i**) extracts from PEO(10%)fb(0.5%) and (**e**,**j**) from HSAm(10%)fb(0.5%) IPNs.

Additionally, the morphology of L929 mouse fibroblasts exposed to the various extracts from the three investigated materials was visualized under phase contrast microscopy (Figure 3). The cells displayed their characteristic spindle-shaped morphology (Figure 3h–j) and were spread through the entire surface of the culture plates reaching near full confluency (Figure 3c–e) after three days of incubation. These data support that the investigated IPN hydrogels are non-toxic to cells. As expected, the cells exposed to the latex extraction vehicle, used as our positive control for cytotoxicity, were spherical, and only a small fraction remained alive and attached to the cell culture plate (Figure 3a,f).

### 2.3. Interactions of Organotypic-Derived Cells with IPN Hydrogels

Organotypic culture was performed to validate the biocompatibility in a more complex situation than the in vitro culture and to assess the behavior of different cell types in contact with the IPN materials. The use of this technique is particularly relevant knowing that the biocompatibility of a specific material is not only dependent on its own characteristics, but has also to be considered within the context in which the material will be used [53].

The overall organotypic results obtained after one week of culture of the different explants in contact with PEO(5%)coHSAm(5%)fb(0.5%) IPNs compared to those obtained with the control (Thermanox^®^, tissue-culture treated coverslips) are summarized in this section.

These data highlight the significant differences in terms of migration, adhesion and proliferation of cells from the corresponding explanted tissues or organs to the IPN hydrogels.

#### 2.3.1. Cell Migration from Tissues to IPN Hydrogels

**Figure 4:**
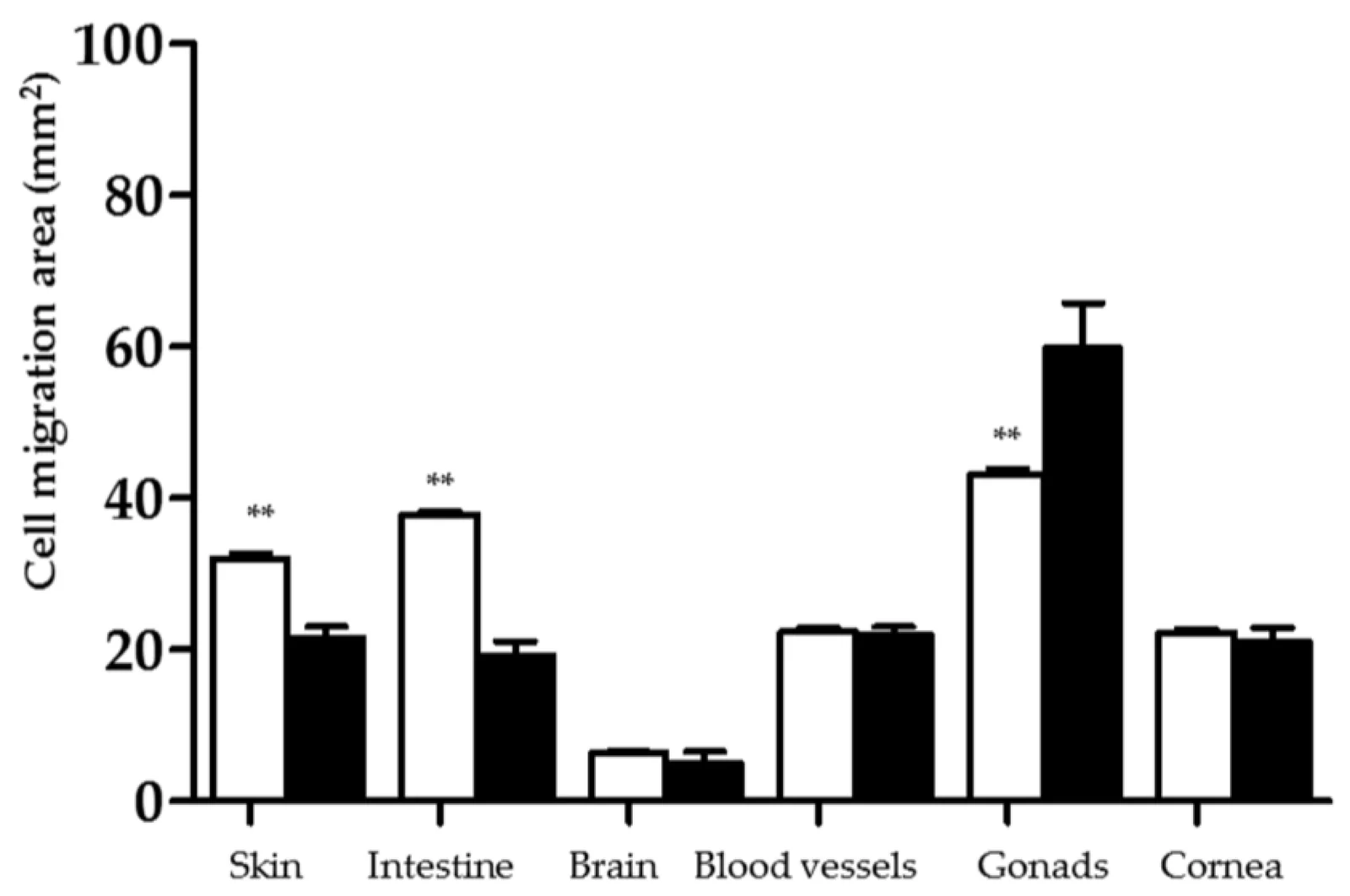
Cell migration assessment (mm^2^) on PEO(5%)coHSAm(5%)fb(0.5%) IPNs (white) and Thermanox^®^ (black) after a week of culture. The surface of cell layers surrounding the explants was determined using ImageJ software on neutral red-stained sample pictures taken with a stereomicroscope fitted with a camera. Data were analyzed using Student’s *t*-test, ** *p* ≤ 0.001 compared to the Thermanox^®^ group.

As cell migration is a defining step essential to numerous biological processes such as skin wound healing [19], it is crucial to study this parameter when characterizing the cell behavior. The cell migration area (expressed in mm^2^) was measured after seven days of culture from the different explants to the IPN hydrogels (Figure 4).

Except for the gonad tissues, our fibrin-based IPNs allowed cell migration similarly to if not better than Thermanox^®^, used as our positive control for cell culture.

Cells from skin and intestine tissues may have more affinity for fibrin than the other cells investigated as they migrated more significantly on PEO(5%)coHSAm(5%)fb(0.5%) IPNs than on Thermanox^®^ (32.07 ± 1.20 mm^2^ vs. 21.65 ± 2.35 mm^2^; 37.87 ± 0.75 mm^2^ vs. 19.28 ± 3.20 mm^2^, respectively). Cells from gonad tissues migrated less on PEO(5%)coHSAm(5%)fb(0.5%) IPNs than on Thermanox^®^ (43.11 ± 1.28 mm^2^ vs. 59.82 ± 10.26 mm^2^). No significant differences were observed between PEO(5%)coHSAm(5%)fb(0.5%) IPNs and Thermanox^®^ for the other explants.

#### 2.3.2. Cell Density Assessment on IPN Hydrogels

**Figure 5:**
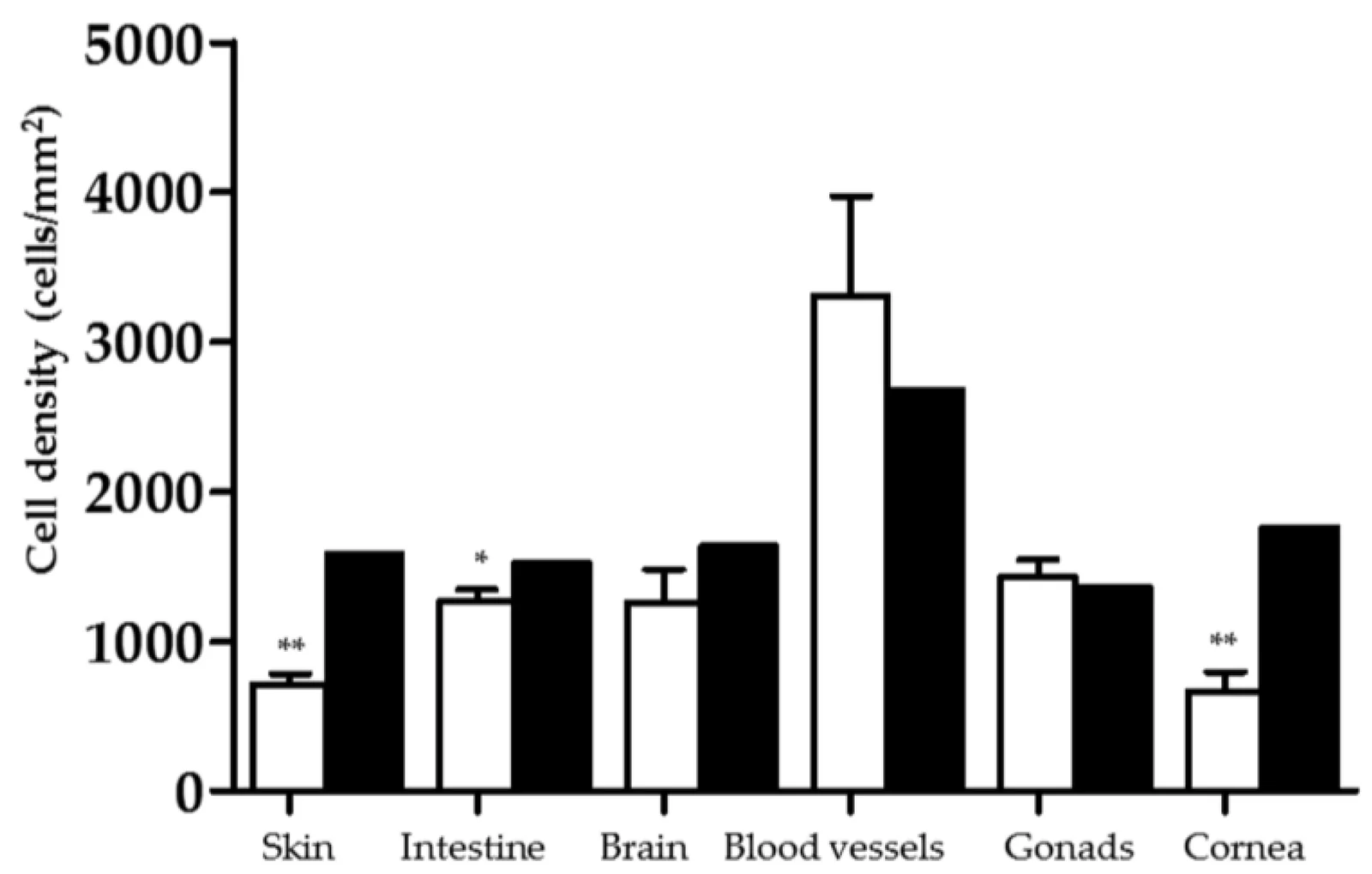
Cell density (cells/mm^2^) on PEO(5%)coHSAm(5%)fb(0.5%) IPNs (white) and Thermanox^®^ (black) after a week of culture. This parameter was calculated after enzymatic dissociation using a trypsin-EDTA in Isoton II electrolyte solution at different time points (5, 10, 20, 30, 60 and 75 min) in physiological conditions (37 °C, 5% CO_2_, 90% humidity). * and ** indicate a significant difference (*p* ≤ 0.05 and *p* ≤ 0.001, respectively) compared to the Thermanox^®^ control (Student’s *t*-test).

The cell density (cell/mm^2^) constitutes a critical parameter and starting point to define the cell proliferation. As shown in Figure 5, the skin (705 ± 131 cell/mm^2^ vs. 1586 ± 0.01 cell/mm^2^), intestine (1266 ± 138 cell/mm^2^ vs. 1522 ± 0.01 cell/mm^2^) and cornea (661 ± 229 cell/mm^2^ vs. 1757 ± 0.01 cell/mm^2^) cell densities were significantly decreased on PEO(5%)coHSAm(5%)fb(0.5%) IPNs compared to on Thermanox^®^. Gonad, brain and blood vessel cell densities were not significantly different between PEO(5%)coHSAm(5%)fb(0.5%) IPNs and Thermanox^®^ (1427 ± 205 cell/mm^2^ vs. 1364 ± 0.01 cell/mm^2^; 1258 ± 390 cell/mm^2^ vs. 1634 ± 0.01 cell/mm^2^; 3473 ± 529 cell/mm^2^ vs. 2671 ± 0.01 cell/mm^2^, respectively).

#### 2.3.3. Cell Adhesion Assessment on IPN Hydrogels

**Figure 6:**
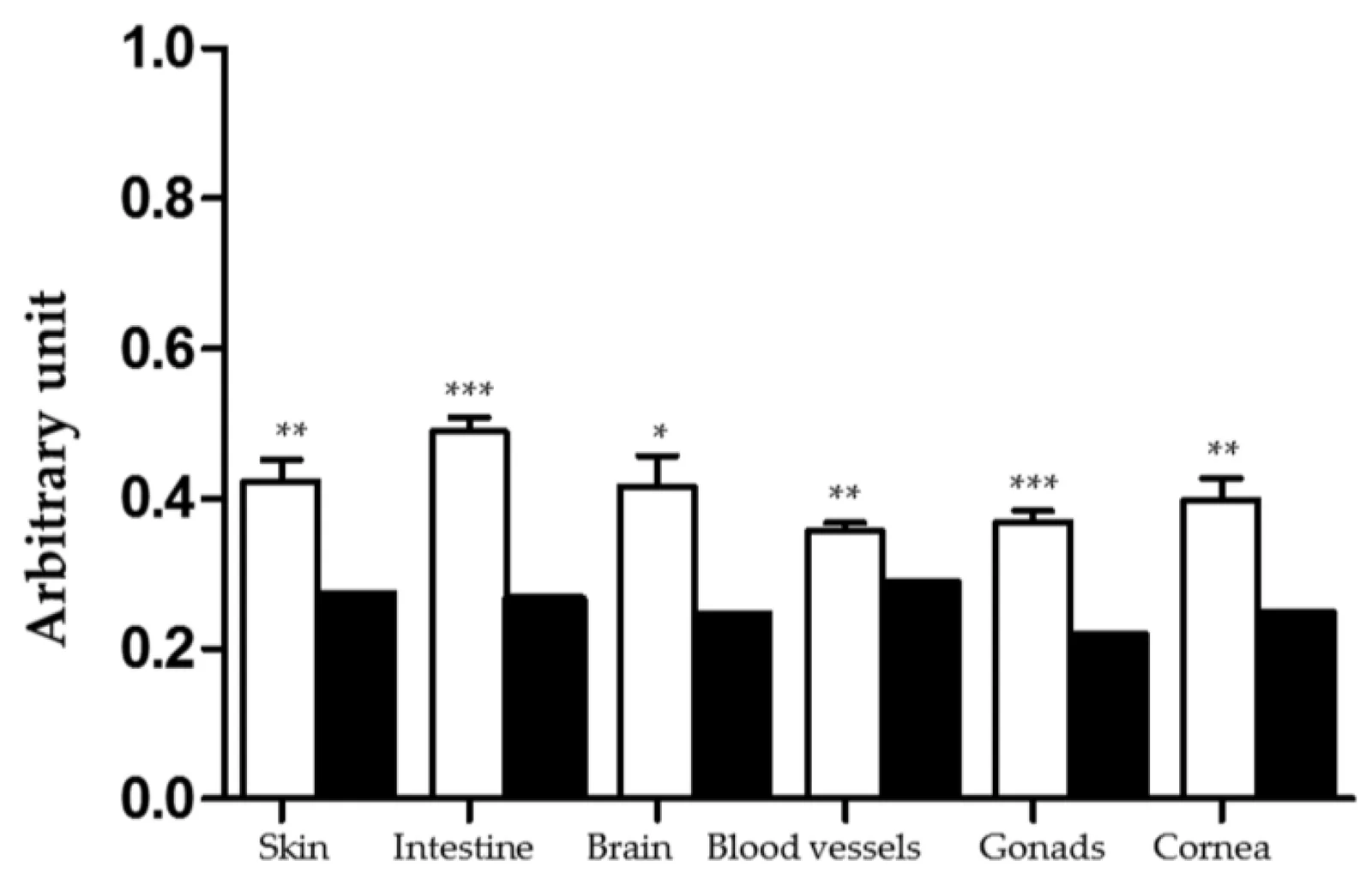
Cell adhesion (arbitrary units) on PEO(5%)coHSAm(5%)fb(0.5%) IPNs (white) and Thermanox^®^ (black) after seven days of culture. The dissociation curve (percent of detached cells as a function of time) was plotted after enzymatic dissociation. An index representative of the inverse of the area between the dissociation enzymatic curve and the x-axis was calculated and used to express cell adhesion. Data, compared to the Thermanox^®^ group, were analyzed using Student’s *t*-test, * *p* ≤ 0.05, ** *p* ≤ 0.001 and *** *p* ≤ 0.0001 compared to the Thermanox^®^ group.

Cell adhesion is one of the most relevant characteristic to examine when assessing the biocompatibility of a biomaterial. This parameter is directly related to the surface, composition and the structure of the biomaterial considered [54]. The cell adhesion parameter was measured by using an arbitrary index inversely proportional to the cell detachment kinetics. As depicted in Figure 6, across all the organs and tissues investigated, cell adhesions were increased when compared to our cell culture positive control (Thermanox^®^ group). These data suggest that our fibrin-based IPNs are bioactive as they can attract and retain a large number of cells.

#### 2.3.4. Cell Morphology on IPN Hydrogels

The cell morphology gives us an insight into how the migrated cells from various tissues are interacting with their host IPN matrices.

• Environmental scanning electron microscopy (ESEM) images

**Figure 7:**
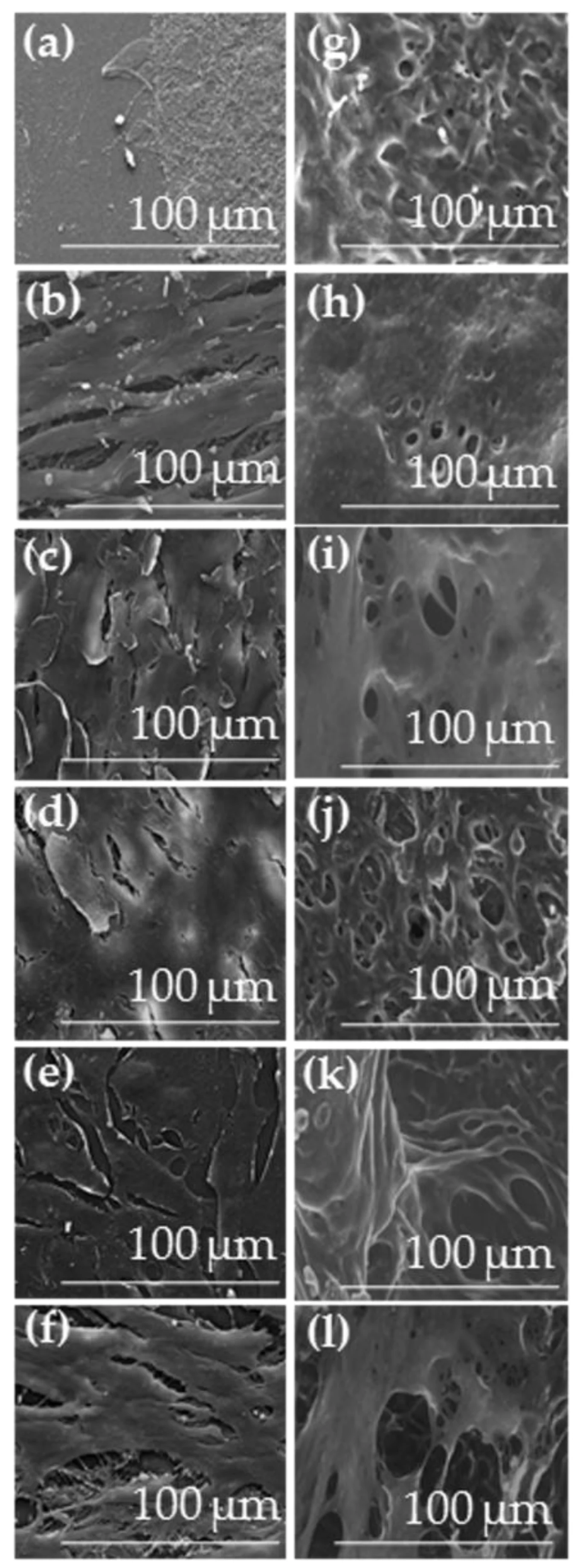
Morphology of cells on IPN hydrogel surfaces after organotypic culture. ESEM images (×1000) of the cells across six different organs/tissues: (**a**,**g**) brain; (**b**,**h**) intestine; (**c**,**i**) skin; (**d**,**j**) blood vessels; (**e**,**k**) cornea; and (**f**,**l**) gonads; cultivated on (**a**–**f**) Thermanox^®^ and (**g**–**l**) PEO(5%)coHSAm(5%)fb(0.5%) IPNs.

Due to their high water content, cell-coated PEO(5%)coHSAm(5%)fb(0.5%) IPNs were observed using ESEM equipped with a Peltier cooling device (Figure 7g–l). Control samples (tissue-culture treated coverslips deposited on explants) were analyzed by scanning electron microscopy (SEM) (Figure 7a–f). The different cell types anchored on the Thermanox^®^ surfaces and adopted their characteristic morphologies such as skin cells in desquamation (Figure 7c), squamous intestinal cells (Figure 7b) or cornea cells (Figure 7f). The cell shapes were more difficult to distinguish on the IPN surfaces due to uneven gel surfaces.

• Confocal laser scanning microscopy (CLSM) images

**Figure 8:**
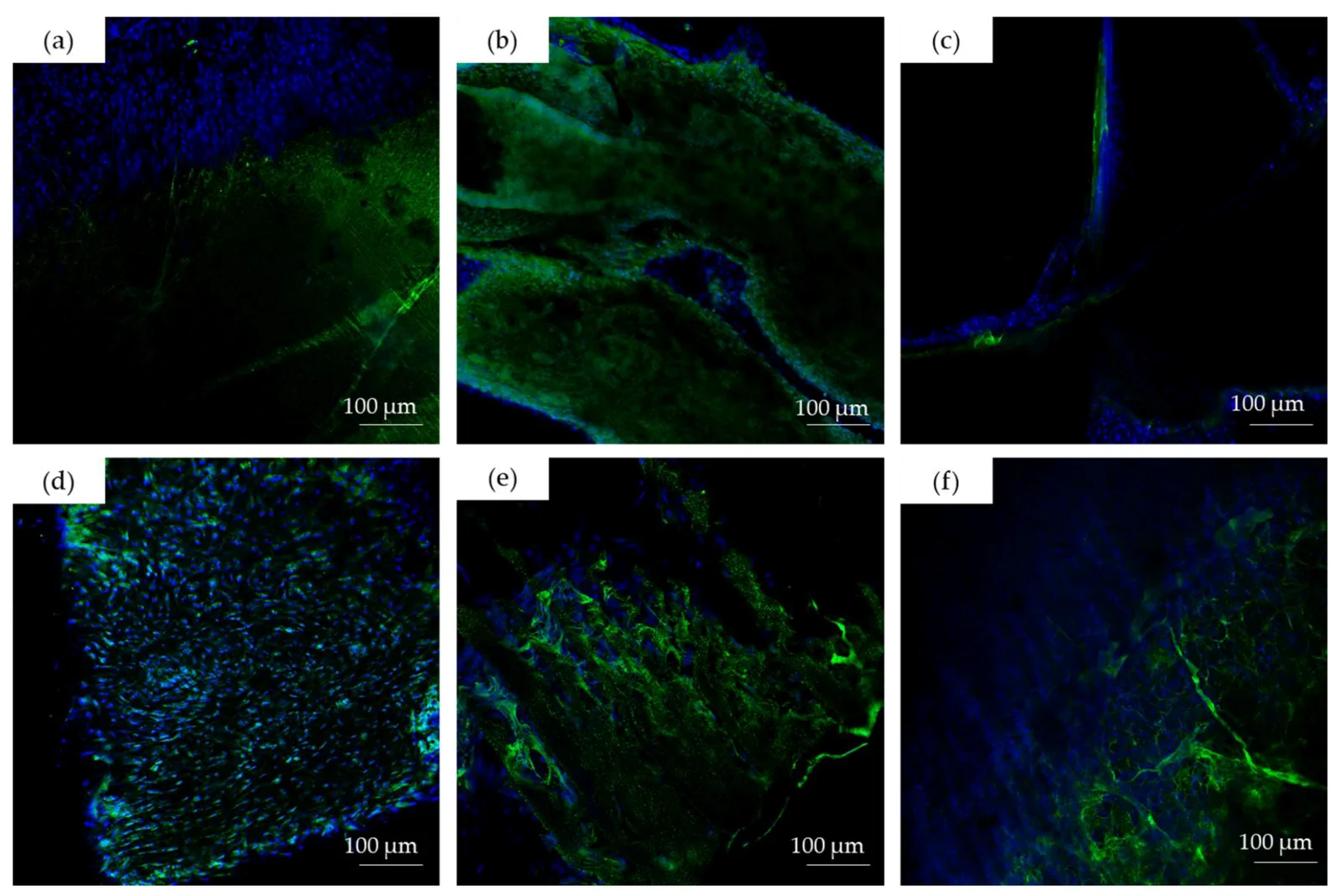
CLSM images (×20) of: (**a**) brain; (**b**) intestine; (**c**) skin; (**d**) blood vessels; (**e**) cornea; and (**f**) gonad cells on hydrogel surfaces after organotypic culture. Cell nuclei (blue) were labeled using Hoechst 33,342 (blue), and the fibrin network (green) was stained using an FITC conjugated to anti-fibrinogen antibody.

To better visualize the cell repartition on the IPN surfaces, immunostaining of the cell nuclei and the fibrin network was performed. Snap CLSM images showed that a large number of cells was attached to the surfaces of our fibrin-containing IPNs (Figure 8).

### 2.4. In Vivo Implantations

**Figure 9:**
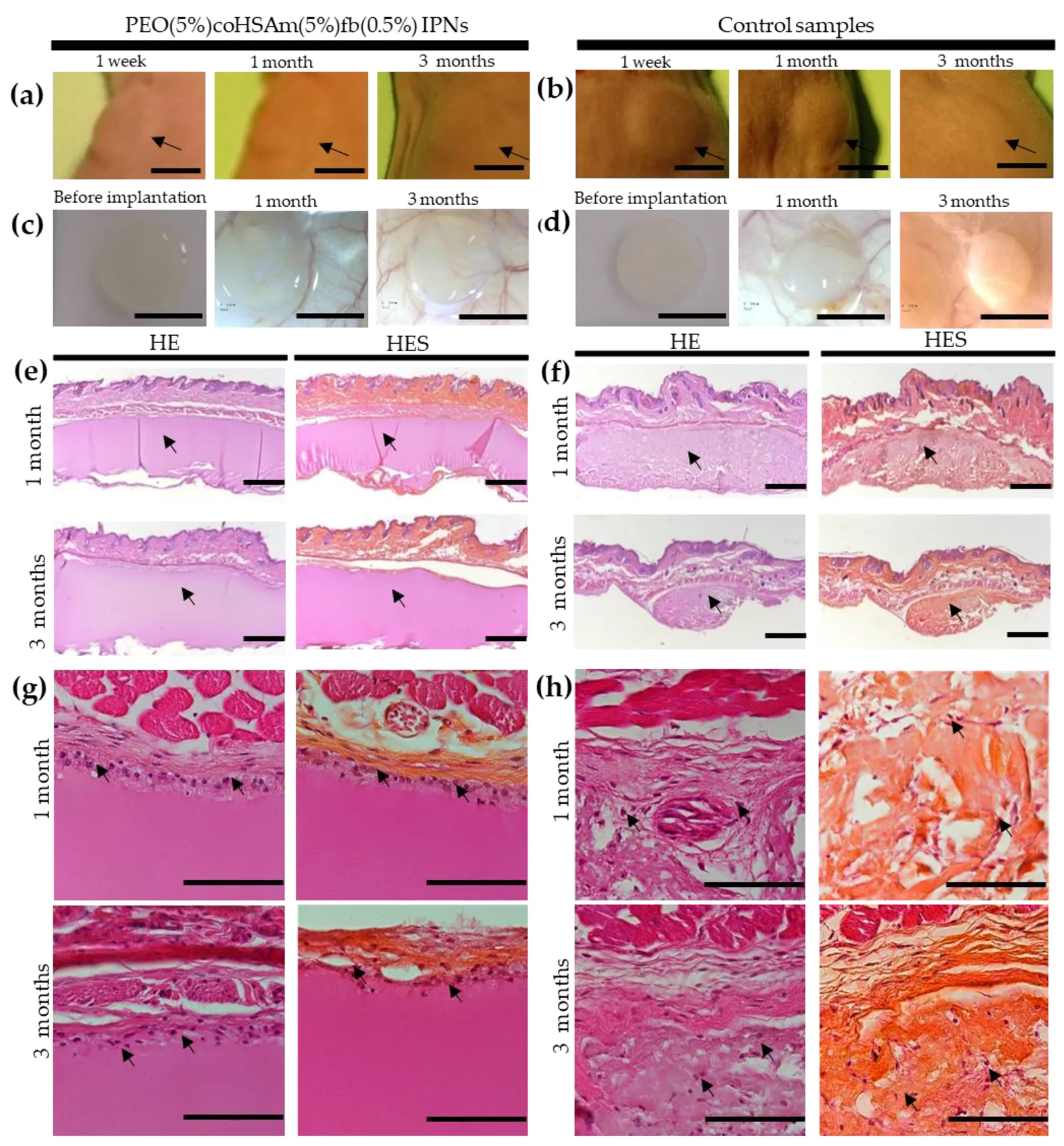
Macroscopic and histologic analyses of PEO(5%)coHSAm(5%)fb(0.5%) IPNs and control collagen-based materials after subcutaneous implantations on nude mice. Nude mice grafted with PEO(5%)coHSAm(5%)fb(0.5%) IPNs (**a**) and control collagen matrices (**b**) after 1 week, 4 weeks and 12 weeks of implantation. Scale bar = 5 mm. Macroscopic images of PEO(5%)coHSAm(5%)fb(0.5%) IPNs (**c**) and control collagen samples (**d**) before implantation, after 4 weeks and 12 weeks of implantation. Scale bar = 5 mm. Histologic analysis pictures of PEO(5%)coHSAm(5%)fb(0.5%) IPNs (**e**,**g**) and control (**f**,**h**) after one month and three months of experiments with hematoxylin eosin staining (HE) and hematoxylin eosin saffron staining (HES). Black arrows depict IPN hydrogels (**e**) and the control collagen samples (**f**), cells on IPN gels within a fibrous capsule (**g**) and cells within collagen scaffolds (**h**). (**e**,**f**) Macroscope images with magnification ×1.6. Scale bar = 500 μm. (**g**,**h**) Microscope images with magnification ×40. Scale bar = 100 μm.

To assess the in vivo biocompatibility, we performed subcutaneous implantations of disk-shaped IPN hydrogels (6 mm of diameter and 1 mm of height) on male nude mice (five weeks old) (Figure 9). We checked the presence of potential irritation and inflammation responses, the cellular infiltration of our biomaterials, the integration of our matrices within the surrounding tissues and the persistence/degradability of our scaffolds after one and three months post implantation. We also implanted commercialized porcine collagen acellular dermis (Meccellis Biotech, La Rochelle, France) and used it as a control for our study.

We could notice signs of wound healing with complete absorption of surgical sutures after one week. Any cutaneous reaction such as erythema, edema and necrosis was observed the days after the implantations for the two implanted groups.

The IPN materials kept their shapes and volumes one month after their implantations (Figure 9a, center image) compared to the collagen control implants (Figure 9b, center image). Their volume slightly decreased after three months, but remained close to the initial volume of the implanted materials (Figure 9a,c, right images), while the overall volume of the collagen samples decreased (Figure 9b,d, right images).

A minimal inflammatory response was observed one and three months (Figure 9e) after the implantation of the PEO(5%)coHSAm(5%)fb(0.5%) IPNs and the control collagen implants (Figure 9f). Macrophagic cells and thin pseudo-fibrous capsules surrounding the implants were observed after one month. The capsules did not thicken after three months.

We noticed that cells did not penetrate within the IPN materials up to three months post implantation (Figure 9g), contrary to the collagen specimens, which had been invaded by cells since the first month (Figure 9h).

## 3. Discussion

In this study, we checked the potentiality of PEO-SAm/fb IPNs to be biomaterials for tissue engineering.

Primarily, we evaluated the biocompatibility of our materials and their capacity to be used as biomaterials in a number of biomedical applications. Assessing the biocompatibility of a new biomaterial is a critical step before its use [55,56]. The MTT cytotoxicity assay is often used to evaluate the biocompatibility as a first screeningand the ISO 10993-5 guidelines proposes to test the in vitro cytotoxicity using an MTT assay on the L929 cell line [56,57,58,59]. Therefore, we performed an MTS assay which is equivalent to the MTT assay on L929 cells with extraction vehicles from our materials. Our preliminary data suggested that our hydrogels were cytocompatible.

These results were validated ex vivo using an organotypic chick embryo model. Organotypic culture is an efficient technique to further evaluate the biocompatibility of biomaterials and to assess simultaneously cell-matrix interactions of different cell types in contact with the investigated biomaterials [60]. This method provides crucial information since biomaterials for tissue engineering need both to prevent inflammatory responses and stimulate specific cellular functions (e.g., adhesion, proliferation, differentiation or migration) [8,17,53,61]. With this technique, we found that skin and intestine cells migrated significantly more on PEO(5%)coHSAm(5%)fb(0.5%) IPNs than on the controls. These results could be related to the key role of fibrin in promoting cell migration during different biological processes [15]. For instance, during skin wound healing, epidermal cells from cut epidermal edges and hair follicles use the fibrin provisional matrix as a template to migrate and cover the wound in a process called reepithelialization [15,17]. Fibrin gels also display good cell migration properties in vitro [15,62,63,64,65].

A quantification of the cell density using organotypic culture showed that skin and intestine cells proliferated less on the tested IPNs than on the controls. Since cell density has been reported to be inversely proportional to cell migration [60], these results could be correlated with the increased migration pattern observed for these particular organs. In addition, gonad cells adhered more on fibrin-based IPNs that on the control materials, but migrated to a lesser extent. Gonad cells such as sperm cells are known to be very mobile [66]. An assumption is that gonad cells may have been physically hindered due to a low gel pore size.

We also investigated the endothelial cell behavior on our matrices. Fibrin is involved in various biological processes including vascularization, angiogenesis and hemostasis [19]. Fibrin hydrogels are thus widely used as scaffolds for cardiac tissue engineering [17,67,68,69,70,71]. In our study, neither the endothelial cell proliferation nor migration were seen to be influenced by the fibrin-based IPNs. However, endothelial cell adhesion, the last parameter investigated with our technique, was significantly enhanced on the tested IPNs compared to the controls. These data indicate that our scaffolds provide a good balance between migration, proliferation and adhesion of endothelial cells, which is suitable for blood vessel reconstruction [60].

Next, the behavior of cornea cells on the fibrin-based IPNs was investigated. The results showed that these cells were more prone to adhere and grow on the IPN hydrogels when compared to the control groups. These findings confirmed previous studies in which fibrin-based matrix was able to support and promote cornea cell growth [72,73,74].

Overall, a significant increase of cell adhesion was seen across the different cell types supporting the potential use of the fibrin-based IPNs as scaffolds for tissue engineering. CLSM images confirmed the role of fibrin to promote the anchorage of cells on our materials as it has excellent adhesion properties [17]. The fibrin network exhibits specific binding sites through which it can interact with integrins and other cell receptors enabling cell adhesion [16,75]. These results highlighted the need to provide biological patterns to the PEO network, which alone displays anti-adhesion properties [34].

To further evaluate the biocompatibility and biointegration of the developed IPN hydrogels within the surrounding tissues, we performed subcutaneous implantations on a nude mice model. Our scaffolds appeared to be well tolerated in vivo with limited infiltration of inflammatory cells within the materials, and only a thin fibrous capsule surrounded the matrices up to three months post implantation. The formation of a fibrous capsule or “shell” after the implantation of a biomaterial [76] is a physiological response to a foreign body, and it has been well reported in the literature [77,78,79]. This capsule maintains the implant position [80] and forms a physical barrier that prevents any further stimulation of inflammation [80].

Additionally, our results indicate that our PEO-containing IPNs are physically stable for several months as they remained nearly intact for the extent of the study. The slow degradation rate of the PEO(5%)coHSAm(5%)fb(0.5%) IPNs may be imparted to non-degradable poly(methyl methacrylate) fragments within the backbone. Further studies should be conducted to study long-term degradation of the fibrin-based IPNs or in order to change the composition of our PEO-HSA/fibrin IPN hydrogels. Various strategies are investigated to fine-tune the biodegradation rates of the fibrin-based IPNs: Increasing the albumin/PEO ratio and decreasing the degree of chemical modification (methacrylation) of albumin.

The in vivo data suggest that our PEO-containing IPNs fulfill the main requirements of soft tissue augmentation fillers as the lack of structural integrity over time and the presence of an immunogenicity are the major limitations of naturally-derived fillers [8,81].

A limited host cellular infiltration was also observed within our IPN hydrogels up to three months post implantation. This lack of cellular invasion within our constructs could be the result of a small pore size combined with a low degree of pore connectivity. Scaffolds with high porosity have been reported to promote cellular infiltration in many biological applications such as dermis [82,83,84], cornea [85], nervous [86], vascular [87] and bone [88] tissue engineering. For this purpose, further modifications in processing, such as the design of a higher porous architecture, will be required to facilitate cell-host integration, neovascularization, mass transport and remodeling essential for tissue regeneration [89].

## 4. Materials and Methods

### 4.1. Materials

Human fibrinogen (341,576) and human thrombin (605,190) were purchased from Calbiochem (Merck, Fontenay-sous-Bois, France). Irgacure 2959 (I2959, 0298913AB) was purchased from Ciba (Bâle, Suisse). Methacrylic acid *N*-hydroxysuccinimide ester (NHS, purity 98%, 730,300), albumin from human serum (purity 98%, A1653), sodium chloride (S7653), calcium chloride (C1016), poly(ethylene glycol) dimethacrylate (average M_n_ = 750, density = 1.1, 437,468), Hoechst 33,342 (14,533) and 2,4,6-trinitrobenzenesulfonic acid solution (TNBS, P2297) were obtained from Sigma (Lezennes, France). Boric acid (1,007,650,050) was purchased from Merck (Fontenay-sous-Bois, France). The CellTiter 96^®^ Aqueous One Solution Cell Proliferation Assay (G3580) was obtained from Promega (Charbonnières-les-Bains, France). Penicillin-streptomycin (15140-122), l-glutamine 1 mM (A2916801), Dulbecco’s Modified Eagle Medium (DMEM, 31885-023) and fetal bovine serum (FBS, 10270-106) were purchased from Life Technologies (Montigny-le-Bretonneux, France). Tissue-culture treated coverslips (Thermanox^®^, Nunc^®^, 628,934) and NHS-Rhodamine (5/6-carboxy-tetramethyl-rhodamine succinimidyl ester) mixed isomer (46,406) were obtained from Thermofisher (Montigny-le-Bretonneux, France). Bactoagar (10,455,513) was purchased from Fisher (Villebon-sur-Yvette, France). HEPES (purity 99%, 172,572,500) was purchased from Acros Organics (Illkirch, France). Rabbit anti-fibrinogen antibodies coupled to fluorescein isothiocyanate (FITC) (F0111-1) was obtained from Dako (Les Ulis, France). Biological implant CELLIS^®^ composed of acellular porcine collagen dermis (CN42F) was obtained from Meccellis Biotech (La Rochelle, France). 5/0 POLIGLECAPRONE 25 Vetsuture^®^ (PGC1CN) was purchased from Noévia (Paris, France).

### 4.2. Functionalization of Serum Albumin with Methacrylate Groups

We modified HSA with methacrylate groups following a previously described procedure [40]; the only change made in the protocol is that the dialysis was carried out against HEPES 0.01 M at pH 7.4 instead of Tris buffer. As found previously using a TNBS assay [40], approximatively 67% of ε-amine from lysine residues available in HSA were modified with methacrylate groups.

### 4.3. Hydrogel Syntheses

A schematic of the fibrin/PEO-SAm IPN synthesis is shown in Figure 1. They were synthesized according to previously described procedures [39,40] adapted to these materials.

An IPN formulation referenced as PEO(5%)coHSAm(5%)fb(0.5%) was synthetized by mixing HSAm 5% (*w*/*v*), human fibrinogen 0.5% (*w*/*v*), PEGDM 5% (*v*/*v*) and human thrombin 0.2 U/mL in a buffer solution consisting of NaCl 0.15 M, CaCl_2_ 0.02 M, HEPES 0.01 M pH 7.4 and the photoinitiator, I2959. Two concentrations of I2959 were used: 0.042% (*w*/*v*) (materials synthetized for organotypic culture) and an optimized concentration of 0.25% (*w*/*v*) (materials synthetized for cytotoxic assays and in vivo experiments).

Other materials used as controls for cytotoxic assays were synthetized using the same protocol: HSAm was replaced by PEGDM for PEO(10%)fb(0.5%) IPNs and PEGDM was replaced by HSAm for HSAm(10%)fb(0.5%) IPNs.

Once all of the prepolymer solutions were prepared, they were transferred into a mold consisting of a glass Petri dish on which 1 mm-thick Teflon pieces were sealed. The mold was exposed to UV light (365 nm, power 1.16 mW/cm^2^, distance lamp-samples: 8.4 cm) for 1 h at 37 °C. The distance lamp-samples was adjusted using a sensor (PowerMax PS19Q, Coherent Inc., Santa Clara, CA, USA) and the PowerMax PC software (version 1.0).

### 4.4. Biological Characterization

#### 4.4.1. MTS Cytotoxic Assay Following the ISO-10993 Guidelines

The cytotoxicity of PEO(5%)coHSAm(5%)fb(0.5%) IPNs was evaluated in vitro with an MTS cytotoxicity assay following the different steps as described in the ISO-10993 standards (Part 5) [49]. The 3-(4,5-dimethylthiazol-2-yl)-5-(3-carboxymethoxyphenyl)-2-(4-sulfophenyl)-2H-tetrazolium, inner salt (MTS) assay is based on the same principle and required fewer steps than the MTT assay [90,91,92]. Briefly, the MTS tetrazolium compound is converted to a colored soluble formazan product by dehydrogenase enzymes produced in metabolically-active cells. The quantity of formazan product was measured by absorbance at 490 nm and was directly proportional to the number of metabolic active cells in culture.

• Preparation of IPN samples

A total number of samples of 24 PEO(5%)coHSAm(5%)fb(0.5%) IPNs (tested materials), 24 samples of PEO(10%)fb(0.5%) and 24 samples of HSAm(10%)fb(0.5%) control IPNs of 690.8 mm^2^ (total surface area for each sample) and 1 mm of thickness was synthetized under sterile conditions. They were then rinsed 3 times in L929 complete culture medium. Latex samples were used as the positive cytotoxic control. Non-treated cells (exposed to L929 complete culture medium) were used as the negative cytotoxic control.

• Preparation of cell culture

L929 cells from passages 11–18 were used for the MTS assays. Cells were cultured in DMEM supplemented with 10% depleted fetal bovine serum (FBS) 1% l-glutamine and 1% penicillin-streptomycin at 37 °C in a humidified incubator (5% CO_2_, 90% humidity).

• MTS cytotoxic assay protocol

For each assay, L929 cells were seeded into 96-well plates at a density of 1 × 10^5^ cells/mL (100 μL of cellular suspension per well) and maintained in culture for 24 h (5% CO_2_, 37 °C, 90% humidity). A total of 4 samples for each composition (test and control samples) was simultaneously agitated in 9.2 mL of complete culture medium for 1 day (5% CO_2_, 37 °C, 90% humidity) following the surface/volume ratio of 3 cm^2^/mL as described in the ISO-10993 guidelines (Part 12) [93,94]. After one day of culture, the medium was removed from the L929 cells, and 100 μL of the extraction vehicle (the complete medium in which the samples were agitated) was added to each well. A total of 4 different concentrations of extracts (*v*/*v*) (100%, 50%, 10% and 1%) was tested for each material. Cells were then incubated for 1 day (5% CO_2_, 37 °C, 90% humidity). Two-day post cell seeding, MTS cytotoxic assays were performed by adding 20 μL of MTS cytotoxic reagent per well. The plates were incubated for 2 h (5% CO_2_, 37 °C, 90% humidity), and the absorbance at 490 nm was then measured using a 96-well plate reader. All measurements were normalized using non-treated cell absorbance, and the viability was calculated using the following formula:

Viability (%) = (100 × OD_490 nm extract_)/(OD_490 nm non-treated cells_) (1)

OD_490 nm extract_: mean value of the measured optical density obtained with cells treated with the extracts of the test samples. OD_490 nm non-treated cells_: mean value of the measured optical density obtained with non-treated cells.

#### 4.4.2. Organotypic Culture

• Sample preparation

A total number of 240 disk-shaped PEO(5%)coHSAm(5%)fb(0.5%) IPNs (40 samples per organ tested) with diameters of 6 mm and thicknesses of 1.5 mm was prepared in sterile condition. Tissue-culture treated coverslips (Thermanox^®^, Nunc^®^, 628,934) (12 per organs) with a surface area of 1 cm^2^ were used as negative controls of toxicity. Thermanox^®^ has been shown to improve in vitro cell attachment and growth due to its highly hydrophilic surface. Thermanox^®^ tissue coverslips have thus been extensively used as controls for organotypic culture [60,95,96,97,98,99] and in vitro studies [100,101,102]. 

Explant culture skin, intestine and brain fragments were removed from 7-day-old chick embryos, while blood vessels, cornea and gonad fragments were isolated from 14-day-old chick embryos. Each organ isolated was cut into 12 pieces of 2 mm^3^ placed on Petri dishes covered with nutrient medium (38.8% of DMEM, 10% fetal calf serum, 1% l-glutamine 20 mM, 0.15% penicillin-streptomycin and 50% of pre-warmed Bacto agar 1% in Gey’s solution). One piece of IPNs was transferred into each of the explants. The cultures were then incubated for 1 week under physiological conditions (5% CO_2_, 37 °C, 90% humidity).

• Cytocompatibility evaluation

A total of 40 IPN samples for each organ was quantitatively evaluated.

1. Cell migration

After 7 days of culture, samples were stained with a neutral red solution. Pictures of the total cell area were taken using a stereomicroscope equipped with a digital camera (CANON PowerShot S70, Courbevoie, France). Then, the migration area corresponding to the total area of the cell layer minus the initial explant area was measured using ImageJ software (version 1.6.0).

2. Cell proliferation

For the cell proliferation assay, cells were treated with a 0.025% trypsin-EDTA in Isoton II electrolyte solution and incubated at 37 °C (5% CO_2_, 90% humidity). After 5, 10, 20, 30, 60 and 75 min, dissociated cells were counted using a cell counter (Beckman Coulter, Villepinte, France). During the last fifteen minutes, 0.25% trypsin-EDTA solution was used to detach the remaining cells.

3. Cell adhesion

The percent of detached cells as a function of time was calculated after enzymatic dissociation. Based on previous published protocols [50,51], we calculated an index representative of the cell adhesion. This index is inversely proportional to the area under the cell enzymatic dissociation curve. The index values are ranged from 0–1. The higher the index is, the higher the cell adhesion is.

• Surface characterization of gels in contact with explants

Control samples (Thermanox^®^ coverslips on explants) were first fixed with a Rembaum buffer (glutaraldehyde 3% in phosphate buffer, pH 7.4) for 1 h; water was replaced gradually with ethanol, dehydrated via critical point drying (Polaron Instrument, Nottingham, UK), and then sputter-coated with gold (Polaron). The samples were then examined by scanning electron microscopy (SEM) (XL 30-ESEM^®^ FEG, Philips, Eindhoven, The Netherlands). The PEO(5%)coHSAm(5%)fb(0.5%) IPN hydrogels in contact with the explants were also examined in their hydrated state by environmental scanning electron microscopy (ESEM) (XL 30-ESEM^®^ FEG, Philips, Eindhoven, The Netherlands).

• Cell repartition at the interface of hydrogels in contact with explants

The PEO(5%)coHSAm(5%)fb(0.5%) IPN hydrogels were fixed with a Rembaum buffer. The materials were blocked in phosphate buffered saline (PBS) containing 3% (*w*/*v*) BSA for 3 h. The fibrin network was then labeled with FITC-labeled anti-fibrin antibody (1/100) in PBS-BSA 1% buffer for 3 h at 37 °C. Nuclei staining of the cells that had migrated from the different explants to the hydrogels was performed using Hoechst 33,342 (2 μg/mL) for 1 h. The materials were then rinsed three times with PBS before visualization using Confocal Laser Scanning Microscopy (CLSM) (Zeiss LSM 410 invert, Oberkochen, Germany). Snap images of the hydrogel surfaces were taken at magnification ×20.

#### 4.4.3. Subcutaneous Implantations

• Animal experimental procedure

All grafting experiments were performed under an animal protocol approved by the Animal Care and Ethics Committee of Picardy (National Reference Number 96, CREMEAP). Five-week-old male nude mice (RjOrl: NMRI-Foxn1nu/Foxn1nu, *n* = 20) purchased from JANVIER LABS were used for this study. Nude mice have been already used to study in vivo biocompatibility since they are able to mount an inflammatory response [83,103] and for soft tissue augmentation applications [103,104,105,106]. The animals were housed in a room with regulated airflow, controlled temperature, humidity and light. The mice were acclimated for 7 days before experiments. Before the subcutaneous implantations, we divided the mice into 2 groups depending on the type of matrices to be implanted: PEO(5%)coHSAm(5%)fb(0.5%) IPNs and porcine collagen acellular matrices used as controls (CN42F, Meccellis Biotech, La Rochelle, France). These dermal matrices are commercialized for various applications such as soft tissue augmentation and skin wounds and thus constitute ideal controls for our in vivo experiments.

Mice were anesthetized with isoflurane (3.5% induction) prior to implantation. Scaffolds of 6 mm in diameter and 1 mm in height were implanted subcutaneously in mice on both sides of the dorsal midline. The wounds were closed using absorbable 5/0 POLIGLECAPRONE 25 Vetsuture^®^ (PGC1CN, Novéia, Paris, France). Animals were sacrificed 4 weeks and 12 weeks post surgery.

• Tissue sample preparation and histology analysis

The scaffolds and the surrounding tissues were harvested from the back of nude mice and placed in 4% neutral buffer formalin for 24 h. The biopsy specimens were then dehydrated using a graded ethanol series (70 °C, 96 °C, 100 °C), embedded in paraffin, sectioned (10 μm thickness), stained either with hematoxylin eosin (HE) or hematoxylin eosin saffron (HES).

### 4.5. Statistical Analysis

All values in the present study were expressed as the mean ± standard deviation. Statistical analysis was performed using GraphPad InStat 5.03 software. Statistical significance for MTS cytotoxicity assays was determined using a one-way ANOVA. The data from the organotypic culture experiment comparing IPN samples to Thermanox^®^ were analyzed using a Student’s paired *t*-test. Differences were considered significant at *p* ≤ 0.05 and indicated in the figures as * (*p* ≤ 0.05), ** (*p* ≤0.001) and *** (*p* ≤ 0.0001).

## 5. Conclusions

In this study, we evaluated the capacity of new advanced IPNs exhibiting the intrinsic biological properties of a fibrin matrix and the mechanical stability of a PEO component as potential biomaterials for tissue engineering. We report that these fibrin-based IPNs are biocompatible using a three-step approach (in vitro, ex vivo and in vivo assays).

Additionally, our findings indicate that these materials promote adhesion, proliferation and migration of several cell types, highlighting their versatility. These data are particularly promising as hydrogels for biomedical applications are designed to be biocompatible, but in many case, failed to promote diverse cellular functions easily.

Looking into the future, the use of these new matrices may be suitable for various biomedical applications including skin wound healing or soft tissue augmentation. Their physical and biological properties could be tailored to extend further their applications.
